# Validation of the Continuum of Care Conceptual Model for Athletic Therapy

**DOI:** 10.1155/2015/391459

**Published:** 2015-07-26

**Authors:** Mark R. Lafave, Dale Butterwick, Breda Eubank

**Affiliations:** ^1^Department of Health and Physical Education, Mount Royal University, 4825 Mount Royal Gate SW, Calgary, AB, Canada T3E 6K6; ^2^Faculty of Kinesiology, University of Calgary, 2400 University Drive NW, Calgary, AB, Canada T2N 1N4

## Abstract

Utilization of conceptual models in field-based emergency care currently borrows from existing standards of medical and paramedical professions. The purpose of this study was to develop and validate a comprehensive conceptual model that could account for injuries ranging from nonurgent to catastrophic events including events that do not follow traditional medical or prehospital care protocols. The conceptual model should represent the continuum of care from the time of initial injury spanning to an athlete's return to participation in their sport. Finally, the conceptual model should accommodate both novices and experts in the AT profession. This paper chronicles the content validation steps of the Continuum of Care Conceptual Model for Athletic Therapy (CCCM-AT). The stages of model development were domain and item generation, content expert validation using a three-stage modified Ebel procedure, and pilot testing. Only the final stage of the modified Ebel procedure reached a priori 80% consensus on three domains of interest: (1) heading descriptors; (2) the order of the model; (3) the conceptual model as a whole. Future research is required to test the use of the CCCM-AT in order to understand its efficacy in teaching and practice within the AT discipline.

## 1. Introduction

Injury is common in sport. Specifically, sport-related injuries have been ranked as second only to home and leisure accidents for injury occurrence and third in terms of severity after traffic accidents and violence [1]. The spectrum of injuries that arise can range from catastrophic events to those that are less urgent. This spectrum of events results in the need for a structured injury care model that can be used to manage sport injuries regardless of severity.

Athletic therapists/trainers (i.e., ATs herein) are professional caregivers that are often hired to provide on-site care in the management of sport-related injuries, which is a key competency for working in a field environment [2]. ATs must fulfill a spectrum of competencies that begin when an injury is often witnessed and end when an athlete safely returns to participation.

ATs often adopt emergency care protocols and standards of care for injured athletes from existing standards of medical and paramedical professions [3–6].

The adoption of emergency care protocols for catastrophic and urgent conditions (such as managing an unconscious athlete) seems quite appropriate for the AT profession since there are no specialized standards or expectations for patient care with these conditions. However, anecdotal experiences suggest that the majority of injuries managed by ATs are nonurgent and non-life-threatening (e.g., sprains, strains, and contusions), thus requiring a different paradigm of assessment, management, and treatment [4]. The care provided to an athlete who suffered prolonged loss of consciousness as compared to one who has a first degree ankle sprain would require evaluation and management strategies that are quite different. The former would require the AT to call emergency medical services (EMS) and to evaluate, assess, immobilize, and stabilize the athlete until assistance arrives. The AT may also need to package the athlete in preparation for transportation to the hospital due to the urgent nature of an unconscious athlete. In the ankle sprain example, the evaluation and management are not as clearly defined using emergency care models found in the literature. If an AT were to follow the existing emergency care model to care for a sprained ankle, the AT would need to call emergency medical services, immobilize the athlete, and wait until EMS arrived. This is not likely the most practical nor ideal way to manage injuries of this calibre. ATs are trained to evaluate the type and severity of injuries and are competent to recognize and manage injuries on-field accordingly. Therefore, a model of care must be able to allow for flexibility across the spectrum of injuries; support decision making from the time the AT begins their evaluation to the point where they decide on the best management protocol; and make use of the skill-set of the caregiver. Currently, there are no such models that have strong levels of evidence that exist in the literature for relatively simple neuromusculoskeletal injuries.

ATs are often faced with a number of challenges and variables associated with managing injuries at both ends of this injury severity spectrum in a real-life environment. Expert ATs should be able to handle these challenges and variables proficiently. However, novice or student ATs learning this material for the first time may require a tool such as a conceptual model to facilitate the learning process. Conceptual models are often used for teaching novice learners complex concepts, which ensures that their interactions are accurate, consistent, and complete [7]. A Continuum of Care Conceptual Model for Athletic Therapy (CCCM-AT) may assist the complex relationship between knowledge and the ability to apply knowledge appropriately.

There are a number of definitions of conceptual models in the literature. Mayer defined a conceptual model as “words or diagrams that are intended to help learners build mental models of the system being studied: a conceptual model highlights the major objects and actions in a system as well as causal relations among them” [8, page 43]. Greca and Moreira defined a conceptual model as “an external representation created by researcher, teachers, engineers, and so forth, that facilitates the comprehension or the teaching of systems” [9, page 5]. Conceptual models have been employed successfully to facilitate learning across a variety of disciplines: (a) computer programming content [10]; (b) electricity principles [11]; (c) nursing critical thinking skills [12, 13]; (d) accounting principles [14]; (e) athletic training therapeutic modality decision making [15].

Although conceptual models are popular outside of the AT discipline, there does not appear to be a conceptual model in the literature that comprehensively represents the full spectrum of nonurgent to urgent conditions within the AT discipline. There were some linear models that outlined the primary and secondary survey comprehensively [4, 6, 16, 17] and one circular model that outlined an interdisciplinary and generic approach to care [18]. None of the models, in their entirety, were applicable to the full AT scope of practice.

The purpose of this study was to develop and validate a comprehensive conceptual model that could account for injuries ranging from nonurgent to catastrophic events including events that do not follow traditional medical or prehospital care protocols. The conceptual model should provide a framework for the management of injuries by an AT regardless of severity.

## 2. Methods

### 2.1. Content Validation of the Conceptual Model

Traditionally, content validation processes such as the modified Ebel procedure have been employed to establish content validity of a measurement tool or instrument [19–21]. In the current study, a modified Ebel procedure was employed to establish the heading descriptors in the Continuum of Care Conceptual Model for Athletic Therapy/Training (CCCM-AT). The modified Ebel procedure consists of three stages: (1) simple validation; (2) initial validation; (3) final consensus or removal of items for validation if necessary [22–24]. The first stage consisted of simple validation (or face validity). It was a process whereby a local/regional group reviewed the literature, interacted, and conceived the content of the model. Materials from all existing models were used to build an AT-specific model of health care delivery as part of the simple validation process [4, 6, 16–18].

The initial CCCM-AT was designed, created, and underwent simple validation with the authors of this paper and four other athletic therapists from the University of Calgary and Mount Royal University. This first version of the CCCM-AT was pilot tested for approximately six months with the ATs who participated in the simple validation process. Pilot testing consisted of ATs working in the field mentally envisioning the headings of the CCCM-AT in conditions across the urgent to nonurgent spectrum.

In the second stage of the modified Ebel procedure, the authors identified a group of experts (*n* = 20) external to the University of Calgary and Mount Royal University. These potential validators were educated in both Canada and the United States.

Potential validators were contacted to confirm interest and availability for the project. The final group of validators (*n* = 11) were self-selected and had expertise in caring for community, collegiate, elite, and professional athletes. Further, they were active educators and examiners in accredited Canadian Athletic Therapists' Association (CATA) programs. This group included 11 certified ATs and one AT/physiotherapist. The sample size of validators was consistent with content validation methodology [25].

The 11 validators were informed about the purpose of the project and sent a marking grid through electronic mail. Validators were asked to rate the importance of keeping each heading descriptor, the order of the model, and the conceptual model as a whole using a marking rubric or importance scale (very important, important, and not important) as part of the second stage. Eighty percent consensus was the target for one of the three categories: very important; important; and not important [22–25]. If that target was achieved, it meant the validators agreed with the content of the conceptual model for use by ATs. If 80% was not achieved, a third and final stage of face-to-face discussion would ensue on the importance of keeping the heading descriptors, the order of the model, and the conceptual model as a whole [22–25]. During the face-to-face discussion each item required 80% consensus for inclusion in the final model.

## 3. Results

**Figure 1:**
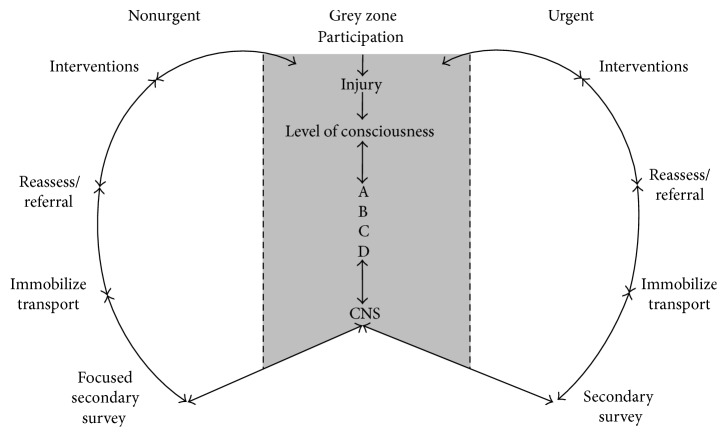
The Continuum of Care Conceptual Model for Athletic Therapy.

The simple validation committee during stage one of the modified Ebel procedure produced a model that was presented to the validators in the second stage. The second stage did not achieve 80% consensus for the three major domains: (1) heading descriptors; (2) the order of the model; and (3) the conceptual model as a whole. However, the third stage's face-to-face discussion did achieve 80% consensus for all three major domains. The final result of the Continuum of Care Conceptual Model for Athletic Therapy/Training (CCCM-AT) is presented in Figure 1. The dramatic change in agreement from stage two to stage three was the result of concrete examples employed to illustrate how the model could be employed.

It was agreed that the CCCM-AT be divided into three major regions: grey, urgent, and nonurgent zones. The face-to-face discussion that ensued and ultimately resulted in agreement for all aspects of the model is central to the results of this paper. Some of the face-to-face discussion included differentiating between the three regions of the model and how they may be different or the same as a traditional emergency care protocol taught in prehospital care educational programs. Below is a synopsis of the discussion that transpired and that is central to the results of this paper.

All injury assessment begins at the top of the grey zone. The grey zone is the time to rapidly gather information about the nature of the injury and assess the patient's vital functions in order to establish which treatment pathway to enter.

Metaphorically, it is grey due to the initial uncertainty about the extent and/or severity of the injury, and the undefined interventions required. For example, if an athlete is unconscious and not breathing, the appropriate management protocol is to activate EMS (i.e., call 911). This is a simple, black and white case to manage, continuing with the color metaphor. However, the majority of other conditions are not as simple, thus requiring greater critical thought on the best management techniques. The traditional prehospital care model employs linear approach to management of conditions where the care provider should complete the ABC's of a primary survey. However, in the AT discipline, we would contend that the evaluation and management should not be simply to complete the linear evaluation outlined in the primary survey. Rather, there may be times for the AT discipline to appropriately and very rapidly (i.e., 5 seconds) evaluate the primary survey and move directly into a focused secondary survey. For example, if a running back in football made a cut and immediately dropped to the ground writhing in pain, grasping their knee* without* contacting an opposition player, an AT may quickly surmise the athlete's airway (A), breathing (B), and circulation (C) are functioning normally. Again, it may be obvious to the AT that the athlete's airway, breathing and circulation are clear because they are screaming in pain and the mechanism of injury did not support external or internal deadly bleeding (D). As a result of the ABC's being clear, the AT may skip to a focused secondary survey or football player's knee injury more quickly. This plan of action is supported with advanced level of emergency care through the International Trauma Life Support (ITLS) for Prehospital Care Providers [26], but not at lower levels of emergency care standards such as those in the Canadian Red Cross [27]. The lower levels of advanced first aid would expect a thorough and complete primary followed by a secondary survey (i.e., head to toe examination). The validator group was given this example and agreed that it accurately represented a common scenario they may be confronted with regularly in their practice. Perhaps more importantly, the validators told the research team that this is the way they currently manage these types of injuries, which further validated the functionality of the model.

In contrast, there may be an athlete who fell to the ground without another player touching them and was lying motionless: no distinct and clear mechanism of injury. An urgent life-threatening injury may be in the AT's mind and a traditional emergency care model should be implemented since there was little explicit information of why this player was lying motionless without obvious cause. In this case, an AT would more carefully and purposefully move through the grey region to ensure the ABC's are evaluated and treated prior to moving into the urgent side of the model (i.e., the right side). Again, the validator group was given this example and all agreed that it could be representative of similar situations where careful and purposeful primary and secondary survey (i.e., not focused) were more appropriate than the first example provided.

These contrasting injuries provide an example of how differently an AT may manage conditions based on seeing the mechanism of injury occur. The examples also illustrate how quickly or slowly one may go through the grey area and move into either the right side (urgent) or left side (nonurgent). To reiterate, if the injury is determined to be urgent, the right side of the conceptual model is the recommended method of management. It incorporates the use of traditional emergency care algorithms found in the literature [4, 6, 16–18]. It also incorporates the notion of a focused secondary survey accepted at the ITLS level of care [26]. The purpose of this model is not to propose any new standards of care if an athlete suffers from an urgent condition. Rather, the purpose of this model is to more accurately represent what transpires in actual practice for ATs.

The utility of the CCCM-AT is both the speed at which one can move from the grey region (centre) to either right or left sides and the speed at which one can focus on the specific injury at hand. The AT scope of practice manages many more non-life-threatening or nonurgent conditions than urgent conditions in everyday practice. This is one of the key differences in the scope of practice between the AT discipline and the prehospital care professional. Prehospital care professionals likely find a bigger balance between urgent and nonurgent conditions, while the AT profession primary deals with nonurgent conditions but has to be prepared to deal with either. When given the option to have a model that captured both urgent and nonurgent conditions so fluidly, the validation group agreed and validated the model provided the arrows were not one directional and linear.

The final but critical aspect of the model that was validated was the use of two-way arrows. It was important to recognize that, at any given stage, athletes (or patients) could suffer an injury that could be classified in the grey, urgent, or nonurgent zones and need to revert one or more steps backwards. The example one validator provided was with the same example of the football running back that was not contacted, thus making the assumption that the injury was nonurgent. However, if that same athlete had the comorbidity of asthma and the athlete went into shock due to pain, it is possible that this athlete's condition could worsen compromising their airway, breathing, and/or circulation. In this case, it would be important to revert back to the evaluation and management procedures outlined in previous, urgent condition steps in the grey zone and quickly move from the left side of the model to the grey zone (center) and then onto the right side of the model. It should be noted that the rest of the CCCM-AT on both the right side and left side after the secondary or focused secondary survey achieved consensus with very little discussion. These examples of the scenarios helped the validators agree and ultimately content validate the CCCM-AT as presented in Figure 1.

## 4. Discussion

In the AT profession, a thorough search of the literature revealed that no conceptual models existed that outlined a continuum of care from the point of initial injury to full return to sport or activity. Additionally, there were no conceptual models that allowed for flexibility in protocols for managing a full spectrum of injury severity. As a result, a new conceptual model was proposed and validated in the current study. The AT discipline is a unique profession whereby the scope of practice may include managing injuries immediately following an injury in a sport or recreational environment. In addition, another unique feature of the AT discipline is the careful guidance and supervision of the athlete after their injury, particularly when the athlete returns to participation in their sport or activity. A conceptual model to help capture this wide scope of practice is important from a teaching, learning, and practice perspective. It can be overwhelming for a novice or student AT to learn, organize and plan the full spectrum of steps from immediate onset of an injury straight through to the eventual return to play due to vast number of steps between those two end points. Placing the entire continuum of care from initial injury to the return to participation into context may be helpful in managing all the steps and variables necessary between each of the major categories outlined in the CCCM-AT, particularly for a novice AT.

Conceptual models have been employed in a number of other disciplines to successfully teach complex concepts and relationships between those concepts.

None of the conceptual models employed in teaching other disciplines reported a similar process of content validation as the process employed in the current study.

However, it seems to be logical to ensure that the conceptual model being proposed actually represents what it is intended to represent: content validity.

The use of conceptual models to teach is supported within a theoretical framework of human cognition. In essence, a novice builds meaning of ideas, concepts, or the steps in a conceptual model by organizing and making connections between those principles [28]. This theory is supported by Ausubel: “If existing cognitive structure is clear, stable and suitably organized, it facilitates the learning and retention of a new subject matter. If it is unstable, ambiguous, disorganized, or chaotically organized, it inhibits learning and retention” [29, page 217]. Theoretically, the conceptual model proposed in the current study is thought to create a “clear, stable, and suitably organized” structure to facilitate learning of the many steps under each major category in the CCCM-AT. It is clear that novices process information differently and move through varying levels of detail differently than experts [30]. A conceptual model, if taught to students early in their learning process, can act like a compass or a guide directing them and contextualizing their learning much like an Advance Organizer or Graphical Organizer [31]. Experts may move through an urgent or nonurgent situation quickly and make, what may appear to a novice, quantum leaps from one conceptual model descriptor to another (i.e., from primary survey to secondary survey) with ease and seemingly little thought [32]. However, an expert has likely lived through a number of experiences that have contributed to their current comfort level with a particular injury or scenario. An expert may be able to negotiate both large steps like those outlined in the CCCM-AT, while also managing the smaller steps that would naturally be embedded under each of those categories. The point of this conceptual model is not to replace those smaller steps under each category listed in the CCCM-AT, but rather to supplement or provide a larger map that helps chart the pathway from the time an athlete injures himself or herself until the point where they return to play. Part of the utility of the CCCM-AT is its simplicity when classifying the complex spectrum of injuries from nonurgent to urgent into one of the three regions.

Schmidt and Boshuizen hypothesized that experts begin to accumulate “illness scripts” over time, which are injuries or conditions that have a similar pattern and thus permit efficiency in an expert's decision making [33, 34]. Fadde suggested that breaking the entire performance down into its pieces and then practicing those pieces may help to hasten the transition from novice to expert [32]. Therefore, it is postulated that this conceptual model could act as a bridge between novices and experts. The model could guide and anchor learning based on a student's perception of the descriptors identified in the CCCM-AT.

One very unique aspect of the CCCM-AT that differentiates it from emergency care models is the concept of a focused secondary survey (FSS) [17, 35]. In the FSS, the urgent pathway of care has been ruled out and a complete secondary survey, including vitals assessment and monitoring, is rarely required. However, advanced first aid education teaches that a head-to-toe examination is necessary to eliminate other sources of injury or potentially life-threatening conditions [27]. This methodology of patient evaluation is one of the differential diagnoses whereby if you eliminate all other things, it must be the one injury or condition remaining that should be the focal point. However, in sport environments or where an AT typically works, it is rare that a condition is ever something more than what the athlete is overtly presenting. If we were to examine this closer using the same example provided in the results above (i.e., the football player who has a suspected noncontact knee injury), in a traditional medical model, the secondary survey would consist of the care provider going through a long, thorough primary survey followed by a head-to-toe examination. It may also require taking vital signs early in the process long before the caregiver ever addressed the athlete's main concern: their knee. To the ATs who were part of the validation group, they thought this type of care for an athlete would be incongruent with the way they practiced or what they wanted to see novices taught or practicing. It is possible for more serious conditions to be present in cases like the athlete's condition regressing due to breathing difficulty, for example. However, in circumstances when complications of a minor injury arise (e.g., shock), the circular nature of the CCCM-AT requires one to return to management strategies as dictated by the grey zone.

The conceptual model in this study has been content validated and needs to undergo future testing and research to determine its efficacy in learning the principles of field-based care in the AT discipline. Research on other conceptual models across other disciplines may provide a foundation for the thesis that this model will help to facilitate learning. Seel demonstrated that students learned economic concepts better when they were exposed to a conceptual model as compared to those students who were not exposed to the conceptual model [36]. Kern achieved more success with teaching an important accounting principle to students using a conceptual model when compared to students who did not use a conceptual model [14]. The question remains whether the results from other disciplines who have had success in teaching using conceptual models are generalizable to the AT discipline and this new model.

## 5. Summary

The athletic therapy discipline is unique with a combination of both clinical and field practice. The profession may be one of the few that is required to manage an athletic injury at both ends of an injury continuum: from the initial injury to return to participation. There were no current models of care in the literature that outlined this unique scope of practice across the full spectrum. The current study adopted the relevant prehospital care professionals' scope of practice and merged it with the AT discipline and scope of practice.

Generally, conceptual models have not been content validated prior to testing their efficacy in teaching and learning. Considering that no models in the AT discipline existed, it seemed appropriate to go through this process prior to testing the conceptual model in an educational environment. Employing conceptual models to teach in other disciplines such as basic science, computer information systems, nursing, accounting, and economics has been successfully implemented. Future research should employ the use of the CCCM-AT to understand its efficacy in teaching within the AT discipline.

## References

[1] Dekker R., Kingma J., Groothoff J. W., Eisma W. H., Duis H. J. T. (2000). Measurement of severity of sports injuries: an epidemiological study. *Clinical Rehabilitation*.

[2] Canadian Athletic Therapists Association

[3] Browner B. D., Pollak A. N., Gupton C. L. (2008). *Emergency Care and Transportation of the Sick and Injured*.

[4] Prentice W. (2009). On the field acute care and emergency procedures. *Arnheim's Principles of Athletic Training: A Competency-Based Approach*.

[5] Rehberg R. (1998). *On Location*.

[6] Starkey C., Brown S. D., Ryan J. (2010). *Examination of Orthopedic and Athletic Injuries*.

[7] Wu C., Dale N. B., Bethel L. J. (1998). Conceptual models and cognitive learning styles in teaching recursion. *ACM SIGCSE Bulletin*.

[8] Mayer R. (1989). Models for understanding. *Review of Educational Research*.

[9] Greca I. M., Moreira M. A. (2000). Mental models, conceptual models, and modelling. *International Journal of Science Education*.

[10] Bayman P., Mayer R. E. (1988). Using conceptual models to teach BASIC computer programming. *Journal of Educational Psychology*.

[11] Royer J. M., Cable G. W. (1976). Illustrations, analogies, and facilitative transfer in prose learning. *Journal of Educational Psychology*.

[12] Desanto-Madeya S. (2007). Using case studies based on a nursing conceptual model to teach medical-surgical nursing. *Nursing Science Quarterly*.

[13] Huang Y.-C., Chen H.-H., Yeh M.-L., Chung Y.-C. (2012). Case studies combined with or without concept maps improve critical thinking in hospital-based nurses: a randomized-controlled trial. *International Journal of Nursing Studies*.

[14] Kern B. B. (2002). Enhancing accounting students' problem-solving skills: the use of a hands-on conceptual model in an active learning environment. *Accounting Education*.

[15] Starkey C. (2013). *Therapeutic Modalities*.

[16] Magee D. (2008). *Orthopedic Physical Assessment*.

[17] National Association of Emergency Medical Technicians (2011). *PHTLS, Basic and Advanced Prehospital Trauma Life Support*.

[18] Miller M., Berry D. (2011). *Emergency Trauma Management for Athletic Trainers*.

[19] Cantor J. A. (1989). A validation of Ebel's method for performance standard setting through its application with comparison approaches to a selected criterion-referenced test. *Educational and Psychological Measurement*.

[20] Ebel R., Frisbie D. (1986). *Essentials of Educational Measurement*.

[21] Violato C., Marini A., McDougall D. (1998). *Assessment of Classroom Learning*.

[22] Butterwick D. J., Paskevich D. M., Lagumen N. G., Vallevand A. L. C., Lafave M. R. (2006). The development of content-valid technical skill assessment instruments for athletic taping skills. *Journal of Allied Health*.

[23] Lafave M. R., Butterwick D. J., Lau B., Murray R., Freeman T. (2013). Content validation of the rodeo SCAT. *International Journal of Sports Medicine*.

[24] Lafave M., Katz L., Butterwick D. (2008). Development of a content-valid standardized orthopedic assessment tool (SOAT). *Advances in Health Sciences Education*.

[25] Lynn M. R. (1986). Determination and quantification of content validity. *Nursing Research*.

[26] Campbell J. E. (2013). *International Trauma Life Support for Prehospital Care Providers*.

[27] Canadian Red Cross Society (2012). *Canadian Red Cross Emergency Care*.

[28] Ifenthaler D., Masduki I., Seel N. M. (2011). The mystery of cognitive structure and how we can detect it: tracking the development of cognitive structures over time. *Instructional Science*.

[29] Ausubel D. (1963). Cognitive structure and the facilitation of meaningful verbal learning. *Journal of Teacher Education*.

[30] Carraccio C. L., Benson B. J., Nixon L. J., Derstine P. L. (2008). From the educational bench to the clinical bedside: translating the dreyfus developmental model to the learning of clinical skills. *Academic Medicine*.

[31] Alba J. W., Hasher L. (1983). Is memory schematic?. *Psychological Bulletin*.

[32] Fadde P. Look 'ma, no hands: part-task training of perceptual-cognitive skills to accelerate psychomotor expertise.

[33] Schmidt H. G., Norman G. R., Boshuizen H. P. A. (1990). A cognitive perspective on medical expertise: theory and implications. *Academic Medicine*.

[34] Schmidt H. G., Boshuizen H. P. A. (1993). On acquiring expertise in medicine. *Educational Psychology Review*.

[35] Campbell S. M., Roland M. O., Buetow S. A. (2000). Defining quality of care. *Social Science & Medicine*.

[36] Seel N. M. (2001). Epistemology, situated cognition, and mental models: ‘like a bridge over troubled water’. *Instructional Science*.

